# Unfair labour practice on staff in primary health care facilities, North West province, South Africa: A qualitative study

**DOI:** 10.4102/curationis.v45i1.2171

**Published:** 2022-02-24

**Authors:** Maserapelo G. Serapelwane, Eva M. Manyedi

**Affiliations:** 1 School of Nursing, Faculty of Health Sciences, North-West University, Mmabatho, South Africa

**Keywords:** unfair labour practice, primary health care facilities, operational managers, North West province, South Africa

## Abstract

**Background:**

Unfair labour practices on staff is a worldwide concern which creates conflicts and disharmony among health workers in the workplace. It is found that, nursing staff members are unfairly treated without valid reasons in primary health care (PHC) facilities and predominantly in the developing countries and South Africa is not an exception.

**Objectives:**

The purpose of the study was to explore and describe the experiences of operational managers regarding unfair labour practices on staff by their local health area managers, and describe the perceptions of operational managers towards such treatment.

**Method:**

A qualitative, descriptive, exploratory and contextual research approach was considered appropriate for the study. The population of the study comprised operational managers working in PHC facilities in the North West province, South Africa. Purposive sampling was used to select participants for the study and focus group interviews used to interview 23 operational managers. Ethical measures were applied throughout the study.

**Results:**

The six phases of thematic analysis were used to analyse the data collected for the study. Two themes that emerged are experiences of factors related to unfair labour practices in the PHC facilities and the perceptions regarding how to improve their working conditions. The categories that were found in the first themes were favouritism and discrimination. In the second theme, in-service training and transparency regarding staff training and development emerged. Recommendations comprised, among others, training on the concepts of equality in the workplace, and reinforcement of transparency regarding granting of study leave and attending workshops.

**Conclusion:**

Operational managers in the PHC facilities experienced unfair labour practices as evidenced by favouritism and discrimination.

## Background

Unfair labour practice is a serious challenge in primary health care (PHC) facilities (Akuffo & Kivipold 2018:12; Sroka & Weinhardt 2018:1). Unfair labour practice refers to being treated unfairly, denied other work benefits and better opportunities, while they are afforded to others by the supervisor (Down 2019:1; Traub Law Attorneys 2020:2). Unfair labour practice can also refer to being disliked by one’s supervisor, negatively criticised and denied leave, while approved for others despite providing a reason (Down 2019:1; Traub Law Attorneys 2020:2). Thus, there is a need to explore the experiences of operational managers with regard to unfair labour practice by health area managers in healthcare facilities. The findings of the study can have an influence on issues related to unfair labour practice on staff during supervision and management of PHC facilities. Operational managers in this study refer to professional nurses, responsible for general supervision of staff and management of the PHC facility and a supervisee to a local health area manager.

Studies conducted in the United States of America, Haiti and Canada revealed biased promotions for some frontline nursing managers, despite their hard work, loyalty and high level of education (Akuffo & Kivipold 2018:9; Gray 2019:10). The studies revealed that frontline nursing managers, who deserved to be moved to the top management positions, were denied the opportunity by their supervisors. Instead of being granted top positions, they (frontline managers) were criticised by their immediate supervisors and considered as quiet, meek, antisocial and managers who were not capable of holding senior management positions (Akuffo & Kivipold 2018:9; Gray 2019:10). Thus, promotions to senior management positions were granted to those selected by their immediate supervisors, irrespective of qualities and competencies required for the position (Akuffo & Kivipold 2018:9; Gray 2019:11). The findings of the study conducted in the United States of America showed that operational managers mostly experienced unfair labour practice in PHC facilities.

The studies conducted in Europe and Poland reported unfair labour practice of staff among operational managers from local and foreign countries (Sroka & Weinhardt 2018:8). Operational managers from Poland revealed they were regarded as not good and their work experience and skills not considered for promotion. Thus, incentives and promotions in Poland and parts of Europe were recommended mostly for people from foreign countries, who did not have sufficient skills. It was further revealed that poor health outcomes and lack of implementation of quality improvement plans were associated with inexperienced managers in Poland (Sroka & Weinhardt 2018). In a study conducted in Asia, Tongerlo (2019:1) found that unfair labour practice hindered the effectiveness of service delivery in healthcare facilities because training was provided to a particular group of staff. The reports of lack of implementation of quality improvement plans, and poor health outcomes suggest that, unfair labour practices on staff can have a negative impact on the quality and provision of health services.

Unfair labour practice on staff is also common in the health facilities of developing countries (Chipeta et al. 2016:8; Saleh et al. 2018:7). In Saudi Arabia, 90% of the professional nurses maintained that nursing managers treated subordinates from other countries unfairly, and those from the same country of origin more favourably. The professional nurses further reported that their supervisors tended to be biased when dealing with problems, which caused psychological stress and low work productivity (Saleh et al. 2018:8). A study conducted in Malawi revealed that area managers of local health facilities were polite with some nurses but impolite towards others which was unfair to them. The study also revealed overt favouritism and lack of confidence or trust and unfairness in the allocation of benefits, such as training and car allowances (Chipeta et al. 2016:8). The studies conducted in Saudi Arabia and Malawi suggest that unfair labour practice of staff can have a negative impact on the psychological well-being of nurses.

Based on the researcher’s nursing experience, one of the core responsibilities of a professional nurse is supervision of nursing activities of health facilities. In Gauteng province of South Africa, some of the professional nurses complained that they were treated unfairly (Khamisa et al. 2016:1). It was shown that they (professional nurses) were not given a chance to supervise their subordinates because the nursing managers assigned duties to their close friends (Khamisa et al. 2016:1). The findings in Gauteng suggest that unfairness in delegation by nursing manager existed as professional nurses who were not friends to the manager were not exposed to supervisory duties. In KwaZulu-Natal province, South Africa, operational managers complained about unfair allocation of resources in PHC facilities as the budget did not cater for equipment to be used by community healthcare workers (Akintola & Chikoko 2016:1). The findings of the study suggest that unfair allocation of resources happened in KwaZulu-Natal because projects for community healthcare workers were not considered in the allocation of resources. Another study in the Western Cape province, South Africa, revealed that poor working relationship among members of the staff was associated with unfair labour practice by their immediate supervisors (Roomaney, Steenkamp & Kagee 2017:7). Inability to meet demands for job description, unfair allocation of material resources and poor relationship suggest that unfair labour practice by supervisors can affect the provision of health services. The studies conducted in South Africa revealed little information of published current research that addresses unfair labour practices of staff in PHC facilities, North West province.

Anecdotal evidence reveals that, local health area managers do not treat operational managers equally in the approval of study leave, delegation to night shifts as well as exposure to senior management activities. It was observed that operational managers who were not close friends to the local area managers, were denied opportunities for study leave. Night shifts and monetary incentives were kept for some members of staff, who were always praised by health area managers as hardworking even when the positive appraisal was not because of them. Similarly, Chipeta et al. (2016:4) discovered frontline managers complaining about their senior nursing managers who displayed overt preferences regarding in-service training even when training was not relevant to them. The literature revealed preferential management style as one of poor supervision and management problems that affect the provision of quality care (Saleh et al. 2018:10). The problem is brought about by the fact that operational managers who are dissatisfied with the ill-treatment by their supervisors, withhold their commitment to patient care and, as such, services suffer. A study conducted in Gauteng province, South Africa, exposed nurses who were concerned about unfair labour practice and increased turn over in PHC facilities (Scheffler, Visagie & Schneider 2015:1). According to Scheffler et al. (2015:1), the operational managers who were considered as favourite staff of senior management did not complete tasks, leading to complaints of poor service delivery by community members. A study conducted in North West province exposed senior management in both hospital and PHC facilities giving special treatment towards certain employees (Mothwane & Muller 2020:50). Special treatment impacted negatively on teamwork and retention of staff in the health facilities of North West province (NWP) (Mothwane & Muller 2020:50). Even though unequal treatment of staff is revealed, there is a dearth of literature regarding the unfair labour practice on staff by supervisors in PHC facilities, North West province, South Africa.

## Objectives of the study

The study explored and described the experiences of operational managers regarding unfair labour practice on staff by their local health area managers in the PHC facilities of the North West province. The study further described the perceptions of operational managers towards unfair labour practice.

## Definition of key concepts

A manager is a person who is responsible for supervising and motivating employees, as well as directing the progress of an organisation (Department of Health of Philippines 2012:25; Royal College of Nursing 2013:5). A manager, in this study, is a person who is responsible for supervising and motivating healthcare workers in a specified PHC facility or a local health area in the North West province, South Africa.

An operational manager is a professional nurse responsible for general management and supervision of staff in a PHC facility, and who ensures the implementation of all PHC programmes and provides quality health care service (National Department of Health 2014:10). An operational manager, in this study, is a professional nurse who oversees the administration, management and supervision of all staff in a PHC facility (National Department of Health 2014:10).

A local health area manager is a designated nursing manager and a supervisor who is responsible for monthly supervisory visits of selected primary health facilities in order to support the operational manager in the supervision and management of PHC facilities of NWP (National Department of Health 2014:10).

For the purpose of this study, local health area manager of a local health facility is an appointment nursing manager in a senior management position responsible for regular support of the operational manager in the supervision and management of PHC institutions of NWP.

A supervisor is a leader who is technically knowledgeable, a good communicator, a good decision-maker with good human relations and capable of promoting teamwork (Department of Health of Philippines 2012:25; Royal College of Nursing 2013:5). In this study, a supervisor is an operational manager, and a local health area manager or any designated nursing manager who is responsible to lead the staff members in PHC facilities of the North West province.

Staff is a concept used to describe people working for a specific establishment or on a specific project (Meena et al. 2016). For the purpose of this study, the concept refers to all categories of nurses working in PHC facilities of the North West province.

The PHC is essential healthcare centred on practical, technically sound and socially suitable methods and technology made universally accessible to individuals and families in the community (Rifkin 2018). In this study, PHC refers to the services provided at the clinics of health districts in NWP.

## Research design and methods

A qualitative approach was adopted and the research design is explorative, descriptive and contextual (Creswell & Creswell 2018:257). The chosen approach and design enabled the study to explore and describe the experiences of operational managers with regard to the unfair practices on the staff by their local health area managers in the PHC facilities of the North West province, South Africa.

## Research setting

This study was conducted at the PHC facilities located in both rural and semi-rural areas of NWP. The North West province consists of four districts, each district divided into sub-districts (Municipalities of South Africa 2012:5).

### Research population

The research population consisted of 56 (12 males and 44 females) operational managers working in the PHC facilities of the four sub-districts of NWP (Statistics SA 2016:1).

### Sampling and sample size

Non-probability purposive sampling was used to select the operational managers in the PHC facilities of NWP. Operational managers were included in the study if they had been working in the PHC facilities of the four sub-districts for, at least, one or more years. The operational managers with less than 1-year of work experience or who were not employed in the PHC facilities of the sub-districts of NWP, were excluded from the study. Twenty-three operational managers were selected to participate in the focus group interviews. Each focus group comprised 5–8 participants. Thus, four focus group interviews were conducted and data saturation was limited to each focus group. For ensuring data saturation, the interviews were discontinued after the participants exhausted their input and were repeating the same information. In this regard, the three of the focus group interviews lasted for 45 min to 1 h and one lasted for 2 h 30 min. In terms of the health research ethics, if the interview lasts longer than 45 min, a break is given to the participants to avoid fatigue and emotional harm which was carried out in this case (Department of Health Republic of South Africa 2015:25).

### Data collection

Data was collected from March 2016 to January 2017 in the PHC facilities of the four sub-districts within the NWP. The authors found the information to be still relevant because currently the literature is silent about the experiences of operational managers regarding unfair labour practices on staff by their local health area managers. In addition, the impact of unfair labour practices that include, among others, increased turnover, lack of team work, conflicts in the work place and community complaints regarding poor service delivery is still existing in the four districts of NWP (Mothwane & Muller 2020:50). Furthermore, it is imperative to report the data from this study because it was obtained from the people at the frontline of management of PHC facilities who are conversant and knowledgeable about labour relations issues. Because of its contextual nature, the data is considered still relevant. Moreover, it was not previously published. The focus group interviews were conducted by the researcher in English, and consent to use a tape recorder was requested and obtained from the participants. An interview guide was used to direct discussions with the following two broad questions: *what are your experiences regarding treatment of staff by local health area managers in PHC facilities*? and *how can treatment of staff be improved in PHC facilities?*

Field notes (observational and personal) were written during data collection (Polit & Beck 2018).

### Data analysis

The six phases of thematic data analysis, in accordance with Braun and Clarke (2006) and Creswell (2014) were used to analyse data. Recorded interviews and their transcriptions were used to organise raw data. Thematic coding was used to categorise raw data into smaller parts to identify important themes and categories. Field notes (personal notes and observational notes) recorded during the interviews were also incorporated into the data and analysed. The services of an independent co-coder, with experience in qualitative research, were used to analyse data and a consensus meeting organised to agree on themes. Data was analysed by first reading through all the transcripts and field notes, which enabled the researcher to generate codes. The codes were generated by making a list of ideas that emerged from the raw data. The codes were further analysed until potential themes, categories and sub-categories were identified. Potential themes were reviewed, refined by re-reading codes and comparing themes with the raw data. Finally, themes and categories were described by making a narrative to show the relationship and how the themes relate to the overall data. Lastly, final themes and categories that emerged from the experiences of operational managers with regard to unfair labour treatment of staff by local health area managers and ways to improve were discussed.

### Trustworthiness

The four principles in accordance with Lincoln and Guba (1981) of evaluating trustworthiness of qualitative research were used in the study Krefting (1991). The principles considered in the study were as follows: credibility, confirmability, dependability, and transferability. The strategies followed to achieve the principles of Lincoln and Guba’s model (1981) are truth value, applicability, consistency and neutrality. For the purpose of credibility and truth value, the researcher had a prolonged engagement with the participants, hence interviews lasted about 45 min to 1 h. During the interviews, prolonged engagement was achieved by applying techniques of clarifying, reflection, paraphrasing, follow-up questions to each and every participant. Confirmability was ensured by verbatim transcription of interviews, and re-reading of information in the transcripts and field notes to get a better understanding of what the participants said about the treatment of staff. In order to achieve dependability and neutrality, data was confirmed with an independent co-coder in a consensus meeting. In addition, the promoter of the study double-checked the transcripts to ensure consistency regarding the research questions, objectives and the entire research process. To ensure transferability and applicability of the study, the researcher thoroughly described the study setting, objectives, methods and findings adequately.

### Ethical considerations

An ethical clearance certificate was requested and obtained from the North-West University Faculty of Research Committee, as well as the School of Environmental and Health Sciences – Mafikeng Campus (NWU-00249-15-A9- certificate number). Permission to conduct the study was also requested and obtained from the North West Department of Health, South Africa. Furthermore, permission was requested (in writing) from the managers of the four sub-districts, where this study was conducted. Permission to conduct research in the PHC facilities was also requested and approved in writing by sub-district managers. In order to adhere to the prescripts of the principle of respect for persons, the purpose of the study was thoroughly explained to prospective participants. The participants were told that their participation in this study was voluntary and that they had the right to withdraw from the interviews at any stage without any penalty. The participants who showed interest in the study, were given consent forms to sign before taking part in the interviews. Permission to record the interviews and take field notes was requested and obtained from the participants. The principle of beneficence was adhered to as during the interviews, the participants were thoroughly observed for verbal and non-verbal cues that could be an indication of emotional harm. The principle of beneficence was also adhered to by clarifying to the participants that the information obtained from the interviews will not be divulged to the public against their wish. For the purpose of conforming to the principle of justice, during the recruitment of prospective participants, voluntary participation was emphasised to ensure fairness in selection and treatment.

## Findings of the study

Twenty-three operational managers (3 males and 20 females) aged between 37 years and 48 years participated in the study. The data revealed that 18 operational managers were in permanent positions and five in acting positions. The professional nursing experience of the participants ranged between 8 and 28 years and their supervision experience ranged from 18 months to 12 years. The summary of the findings is presented in Table 1.

**TABLE 1:** Experiences of operational managers regarding unfair labour practices.

Theme	Categories	Sub-categories
Experiences of factors related to unfair labour practice in the PHC facilities.	Favouritism	Close friendship with some operational managers
		Unfairness related to performance management development system
		Unfairness in allocation of resources
	Discrimination	Internal promotions Study leave and management workshops
Perceptions regarding how to improve on issues related to unfair labour practices	In-service training Transparency regarding staff training and development	Professional working relationship Performance management system

*Source*: Serapelwane, M.G. & Manyedi, M.E., 2019, ‘A supportive supervision framework for operational managers in the primary health care facilities of the North-West Province,’ PhD thesis, Faculty of Health Sciences, North-West University, Mafikeng Campus.

PHC, primary health care.

Two broad themes, with their categories and sub-categories emerged from the consensus meeting held with the independent co-coder. In the first theme, the participants expressed experiences of factors related to unfair labour practices on staff and favouritism emerged as a category from the first theme. The three sub-categories which emerged from the category of favouritism are as follows: (1) close friendship with some operational managers; (2) unfairness related to performance management development system; and (3) Unfairness in allocation of resources. In the second theme, perceptions with regard to how to improve on the issues related to unfair labour practices on staff were advanced by participants and discrimination emerged as a category. The sub-categories that emerged from discrimination were as follows: (1) internal staff promotions; and (2) study leave and management workshop. The following section provides a discussion on the themes, categories and sub-categories.

### Theme 1: Experiences with regard to unfair labour practices on staff

The participants indicated that members of staff, including operational members, were not treated equally. The two categories that emerged from the experiences with regard to unfair labour practices were favouritism and discrimination.

### Category 1: Favouritism

Favouritism was highlighted by the participants, resulting from close friendship with some operational managers and unfairness related to the performance management development system. The sub-categories related to favouritism are as discussed in the following subsection.

#### Sub-category 1: Close friendship with some operational managers

It emerged during the interviews that favouritism displayed through close friendship with some operational managers, hampered the continuity of care, as captured by one of the participants during the focus group discussions:

‘These managers are not aware of the mess they are creating with favouritism [*looks angry*]. Now, there is a lot of disharmony between the facility manager of health centre A and clinic B because facility manager of health centre A is aware that this one is a big favourite of a local health area manager, which also affects continuity of care of patients. The same participant maintained as follows: For example, some of the staff of health centres within the local area are not cooperative in receiving clients from smaller clinics because of the close friendship the local health area manager created with some operational managers.’ (P1, male, 46 years old)

Participant P2 reported favouritism of supervisors of some operational managers as follows:

‘Favouritism pulls us down, because even if you try your level best, you will be knowing that only so and so who is a friend to a manager, her clinic will be seen as the best performing **(looks very furious).** Some of us have already developed a don’t care attitude to the general performance and management of the clinic, which also affects the management of clients.’ (P2, female, 39 years old)

Participant P3 indicated as follows:

‘Here, in this sub-district, it is like this neh, facility manager is your friend, you are going to treat this person this way, and this one from that facility is not your friend and you are going to ill-treat her instead of supporting this person and guide this person to say, this is how this thing should be done in the facility you focus on the friendship and gossip, local health area manager gossiped about the mistakes found on patients referred from my facility to the facility of my colleague, who is her favourite.’ (P3, female, 41 years old)

A study conducted in Russia revealed that a supervisee may experience social alleviation and feeling of not being needed in the organisation as a result of a manager who favours certain members of staff (Safina 2015:632). Similar to what was found in Russia, the current study revealed that social alleviation prevailed, as operational managers who were not favoured by their immediate supervisors, were not guided on how the facility should be managed. Local health area managers involved in this study created a close relationship with some operational managers, which is an indication of favouritism. On the other hand, local area managers were involved in talking about operational managers with their close friends, an indication that close friends were treated better than others. Gossiping involved talking about mistakes identified from patients referred from facilities of the participants not favoured by managers, bordering around compromising professional confidentiality. The current study revealed that the operational managers who realised that they were not favoured by their immediate supervisors, developed a ‘don’t care’ attitude, which had a negative impact on the general performance of the facility. In this study, the participants revealed that favouritism caused disharmony in the workplace. The findings of this study are in line with Fu (2015:3), who found that favouritism brings much conflict and frustrations among members of staff.

#### Sub-category 2: Unfairness with regard to performance management development system

Unfairness with regard to reward based on performance management development system (PMDS) emerged as a sub-category as reported by participant P4, captured in the following excerpt:

‘[*With a raised tone*] There are these people … certain facilities whom it will be known in the sub-district that they are the ones who receive PMDS. Irrespective of low performance indicators, even if their facilities are not so perfect, they … those who are not achieving, they get PMDS what is that, it does not make sense really.’ (P4, female, 38 years old)

Participant P5 also maintained as follows:

‘Some of the things are just happening and you wonder why. There was a time we were supposed to submit the PMDS report. Each clinic had to submit to the local health area manager, surprisingly, there will be this person, one of the facility managers who submits her reports directly to the sub-district manager and why, but we do not have to question.’ (P5 female, 43 years old)

Participant P6 added as follows:

‘With this PMDS like I said, when you try to delegate somebody, this person will say, I will not do this because I am not recognised, I am not the one who is known for receiving performance bonus. Sometimes, some people will just say repeated people will get that PMDS, so people will say so and so is the one who is working and will say go and ask Mary to do that because she is the one who is recognised by the managers.’ (P6, female, 37 years old)

One of the key principles of PMDS in South Africa is to enhance organisational effectiveness and efficiency in a supportive, consultative and non-discriminatory manner (Provincial Government RSA 2015:7). The participants reported that the same people in some facilities were receiving PMDS award, irrespective of their poor performance, which is an indication of unfairness. The participants also reported that those who were not receiving performance bonuses were refusing delegation. The study conducted by Kovane and Williams (2015:82) revealed that most respondents were dissatisfied with PMDS bonuses and maintained that they were paid on the basis of friendship. A similar phenomenon was reported in a study conducted in Nairobi, which revealed that job dissatisfaction was associated with inequitable performance appraisal (Schwendimann et al. 2016:8).

#### Sub-category 3: Unfairness in the allocation of resources

Favouritism was also reported with respect to the unfairness in the allocation of resources. Participant P7 indicated as follows:

‘The resources are not allocated equally because what comes to the head of allocators, are individuals. They do not give the facility according to what you have ordered. If facility F has ordered 10, whatever they give them a lot of things, then the remnants whatever that is left, will be what you get because you are not a friend to them.’ (P7, female, 40 years old)

Another participant (P8) maintained as follows:

**‘Eheh** allocation of human resources, you will find that the head count **(meaning monthly statistics)** of the facility G is far low but her staff allocation is bigger than yours. We are not getting equal treatment from our managers.’ (P8, female, 37 years old)

Participant P9 added as follows:

‘Recently, we were allocated computers, I was shocked where I was seated three days ago, the facility that is having less headcount have twice the allocation of computers than us. The same participant maintained as follows: I am trying to ask myself, are they allocating according to professional nurses because even the number of professional nurses are not five.’ (P9, female, 42 years old)

A study conducted in the Eastern Cape, South Africa, revealed that job dissatisfaction was associated with unequal distribution of resources (Sherry 2017; Kheswa 2019:6). In this study, the participants reported that human and material resources were not distributed according to the facility demands, which is an indication of unfair treatment. The current study revealed that the operational managers were frustrated about facilities with less head count that were getting more staff and material resources, such as computers. The findings of this study are in line with those of Makhamara (2017:96), who found that inefficient allocation of human resources increase the level of stress and staff turnover in one of the district hospitals in Kenya.

### Category 2: Discrimination

The discussions revealed that members of staff, including operational managers, were discriminated against by senior managers. The sub-categories that emerged from discrimination (internal staff promotions, granting of study leave and management workshops) are discussed in the following subsection.

#### Sub-category 1: Internal staff promotions

Discrimination related to internal staff promotions emerged as a sub-category during the focus group discussions. Participant P10 told the following:

‘There are people whom we know do not have management and nursing administration qualification, but just because they belong to those who are liked by management, … they are liked [*high tone of voice*]; the very same people are put on assistant nursing mangers’ positions, just recently, I am not saying last year [*angrily*].’ (P10, female, 38 years old)

Participant P11 maintained as follows:

‘Let me tell you, I went for that internal interviews of two assistant nursing managers’ post, and I feel strongly that I passed the interview but because I was discussed, and I do not belong to them, I did not … never get the post [*waving hands and shaking head*] and that one, I will never forget it in my life. They will discuss you and they will take a ridiculous decision about what you qualify for.’ (P11, male, 43 years old)

Participant P12 also highlighted the issue of discrimination with regard to internal staff promotion and maintained as follows:

‘One of them said to me, poor you, you are a hard worker but people hate you, you do not belong to their group therefore, you will not get anything … and understood from what she said that I will not get any promotional post [*angrily and with a sad tone*].’ (P12, female, 45 years old)

According to the American Nurses Association (2018:1), discrimination is the practice of unfairly treating a person or group of people differently from other people or groups of people. The participants involved in this study told that promotions to assistant nursing managers’ positions were awarded to the operational managers who were favoured by senior managers. In the United States (US), it was reported that discrimination was confined to promotion of young members of staff to senior management positions (Fekedulegna et al. 2019:13).

#### Sub-category: Study leave and management workshop

Discrimination related to the way study leave was granted and management workshop was also highlighted during the focus group discussions. Participant P13 maintained as follows:

‘Some people will be taken for study leave, sometimes, they do not take long again, they will go for training again. Somebody is just here in this waiting list.’ (P13, female, 44 years old)

The same phenomenon was reported differently by participant P14 as follows:

‘Some of the EN’s have been applying neh, will apply and they do not go for study leave … you … we will be knowing deeply that there are people who are not supposed to stay on a waiting list.’ (P14, female, 39 years old)

Discrimination related to management workshop was also highlighted by the participants. Participant P15 indicated as follows:

‘There are management workshops that should to be attended by operational managers but now, it made us to realise that we will never be the same to our local health area managers, we are not … [*Interrupted by two group members … angrily*] not treated the same … yes, we are not treated the same.’ (P15, female, 40 years old)

Participant P15 added as follows:

‘Even now, recently, there is this workshop known as MDP [*managers’ development programme*], where operational managers go once a month, three times within two months, somewhere outside work. They have sent only two operational managers, those they regard as better than us, the other one is so inexperienced, then maybe will go after them, what is that it does not make sense. Are we not supposed to orientate new and inexperienced ones? You still have to orientate this person, but this person is taken, she is … is taken to this broad course. It is not discrimination because it is like when you do not belong to them, you do not exist.’ (P15, female, 40 years old)

To address the issue of discrimination, there is a need for equal training and development opportunities to be given to all members of staff irrespective of their social and geographical background (Equality and Human Rights Commission 2015:12). The participants involved in this study reported that some members of staff are granted study leave without waiting for long, while others remained on the waiting list and not granted study leave, despite their requests for such leave. The participants indicated that two operational managers, who were regarded as better than others, were given the opportunity to attend management workshops and those who were not liked, were left behind. The findings of this study are similar to those of Fekedulegna et al. (2019:14), who found lack of equal opportunities in staff training and development in their study.

### Theme 2: Perceptions regarding how to improve on issues related to unfair labour practices

The participants suggested different ways to improve on issues related to unfair labour practices within the PHC facilities of the North West province, South Africa. Thus, there is a need for in-service training and transparency with regard to staff training and development as discussed in the following section and subsections.

### Category 1: In-service training

The participants indicated the need for in-service training with regard to professional working relationships, performance management development system and these are presented as sub-categories in the following subsections.

#### Sub-category 1: Professional working relationship

The participants also highlighted the need for training with regard to professional working relationship, as captured in the excerpt below by participant P16:

‘People need to be taught and taken privately for … training on how to relate with their subordinates in the work environment.’ Another participant maintained as follows: ‘Maybe training will reduce excessive friendship at work.’ (P16, female, 46 years old)

Participant P17 stated as follows:

‘Top managers … a manager acting like, like they are not professionals, they are acting like they are from somewhere from ‘Pick ‘n pay’, now they come to meet with their friends who are feeling better than some of us.’ (P17, female, 43 years old)

The participants indicated that senior management had to be taught on how to display a professional working relationship. The participants further stated that training could reduce excessive friendship in the work environment. The PHC facilities are meeting venues for close friends. Research by Munyewendel, Rispel and Chirwa (2014) revealed the need for senior management to portray a good working relationship towards professional nurses for the improvement of quality care. The findings concur with what was reported by Melo and Neves (2015) because there was 83% of positive correlation between all leadership roles, including professional relationships and supervision satisfaction of nurses in Portugal. The findings of this study are in accordance with those of Munyewendel et al. (2014) and Melo and Neves (2015) who expressed the need to train top managers on professional working relationships.

#### Sub-category 2: Performance management development system

The operational managers involved in the study highlighted the need to include performance management system within in-service training, as captured by participant P18.

‘… and that is why I am saying they do not understand the policy for PMDS and remember we went for training, all categories attended training, except local health area managers.’ (P18, female, 38 years old)

Another participant indicated as follows:

‘To me, PMDS is just a fruitless exercise; it is not serving any purpose because it deals with individuals and they are not objective.’ (P7, female, 40 years old)

Participant P19 highlighted the need for in-service training regarding PMDS and maintained as follows:

‘Performance appraisal is not done appropriately; people pile up problems … up … [*demonstrating with hands*] and now, they wait for performance appraisal so they use performance appraisal to remind you about long-standing facility issues, something must be done about PMDS in the sub-district [*d*], training is required.’ (P19, female, 41 years old)

Participant P20 added as follows:

‘HR should do in-service training to teach people how this is supposed to be done maybe we will have a common understanding.’ (P20, female, 39 years old)

The findings revealed that the area managers of local health facilities were not trained on PMDS. Lack of objectivity also emerged, and the participants maintained they were reminded about long-standing facility issues during performance appraisal. The participants further indicated the need for the human resource department to train the staff on PMDS for them to have a common understanding. There is also the need for supervisors to be technically knowledgeable as they are expected to teach and train supervisees (Hughes 2017). The literature also revealed that a supervisor should possess specialised knowledge and skills regarding leadership and management processes (Hisler 2016:6). The findings of the current study suggest that the senior managers lacked management and leadership skills.

### Category 2: Transparency regarding staff training and development

The participants highlighted the need for transparency regarding staff training and development in the PHC facilities. This was reported by participant P20 as follows:

‘Training let people go for training equally.’ (P20, female, 39 years old)

Participant P21 maintained as follows:

‘For workshops, we must not have certain operational managers taken for this workshop without all being informed of the reasons why others are left out.’ (P21, female, 39 years old)

Participant P22 added ‘Let us have transparency when taking people for training’ (female, 44 years old).

Participant P23 maintained as follows:

‘If this person was to go for training, let us not jump him and take another one because training will boost the morale of staff; let them go accordingly.’ (P23, female, 42 years old)

The participants involved in the current study suggest the need for transparency in training and staff development, which is a requirement in the PHC facilities. It was further revealed in this study that training can boost staff morale if done appropriately. Transparency in the study is supported by what appears in the report of Equality and Human Rights Commission (2015:12), because it is a key element in staff development and training in the workplace.

## Discussion

The purpose of this study was to explore and describe the experiences of operational managers regarding unfair labour practices on staff by local health area managers in the PHC facilities of North West province, South Africa. The description of ways to improve treatment of staff in such facilities was also provided. Favouritism and discrimination were identified as factors related to unequal treatment of staff in the PHC institutions of NWP. Favouritism causes conflicts among operational managers, especially during referral of client. The operational managers indicated that the lack of cooperation in receiving patients from facilities of their colleagues not favoured by local health area managers was because of gossips. The voices addressing the theme of favouritism revealed that, general performance in terms of management of PHC facilities was negatively affected, shown by the ‘do not care’ attitude in the management of the facility, as portrayed by operational managers who were not favoured by their immediate supervisors. The study by Saleh et al. (2018:201) revealed that favouritism towards certain groups of nurses can demotivate persons who are mindful that their equals are given preferential treatment. Favouritism, in this study, was also evident in performance appraisal and allocation of material resources, as the participants indicated lack of objectivity in awarding performance bonus to the staff members. The participants told that performance appraisal and allocation of resources was based on giving priority to members of staff who were close associates of local health area managers and sub-district managers. Acts of favouritism that emerged as close friendship with some members of staff, unfairness related to performance appraisal, and allocation of resources, demonstrate that there was unequal treatment of staff within the PHC institutions of NWP. Another study conducted in NWP also revealed that the quality of performance appraisal was compromised by favouritism within PHC facilities, which further led to conflicts among members of staff (Madlanebana & Peterson 2020:16). The findings of the current study suggest that discrimination occurred in the PHC facilities, as operational managers, who were not liked, and not belonging to the group of close associates, were not promoted to higher management positions, regardless of their relevant qualifications. Furthermore, the findings suggest that study leave, and management workshops, were granted to those who were inexperienced, and liked by senior management of sub-districts, which is an indication of unequal treatment of staff. Similarly, Meng (2018:22) reported that signs of favouritism in the workplace include granting certain employees more development opportunities than others.

The findings of this study demonstrate that in-service training, focusing on professional working relationship and performance management system, is an integral aspect to improve treatment of staff by their supervisors. Emphasis on transparency regarding staff training and development in PHC facilities of NWP emerged clearly from the findings of the study.

## Implications

The findings of this study provided insight on unfair labour practices, as preferential treatment resulted in conflicts and lack of cooperation and team work among members of staff. It was revealed that unequal treatment on staff had a negative impact on the general management and supervision of the PHC facilities in NWP. Improving unfair labour practices by local health area managers and sub-district managers through avoidance of favouritisms and discrimination may bring about harmony and team work among the staff in the PHC facilities of the districts of NWP.

## Limitations of the study

The study was limited to the PHC facilities situated within the four districts of the North West province, South Africa. The time limit for the focus group interviews exceeded 45 min, hence the participants were given resting sessions in between to avoid emotional harm and fatigue.

## Conclusion

Favouritism is a common practice by the area managers of local health facilities; they have close ties with operational managers and other members of staff. Such practice demonstrates unequal treatment within the PHC facilities. Operational managers, who were not close to such area managers, were not guided on how to rectify errors identified in their facilities. Furthermore, continuity of patient care is hampered by the lack of cooperation among small and bigger clinic managers, involved in gossips with local area managers. Close friendship with subordinates suggests these managers lacked qualities of good leadership. It was revealed that the operational managers, who were not favoured by their supervisors, developed a ‘don’t care’ attitude, which ultimately affected the provision and management of PHC services. Unfairness in the allocation of human and material resources is an indication of unfair labour practices. It was revealed that the local area managers lacked knowledge and skills regarding PMDS as long-standing issues were brought in during performance appraisal. In addition, refusal of delegated duties by nurses, who claimed to be not recognised for PMDS, is an indication that unfair labour practice on staff impacted negatively on the provision of PHC services. Discrimination with regard to internal staff promotions, study leave, and management workshops, suggest the need to improve treatment of staff in PHC facilities in the North West province.

### Recommendations

The following recommendations are suggested based on the findings of the study:

### Nursing practice

Nursing managers should be trained on the attributes of a good leader. This can resolve the issue of unfair treatment of staff by the supervisors, which in turn improves professional working relationships in the PHC facilities of the North West province. All members of staff in the PHC facilities of the four districts of the North West province should be trained on PMDS. It is recommended that all managers should be trained on the concepts of maintaining equality in the workplace, with special emphasis on the detrimental effects of discrimination and unfair labour practice. Transparency with specific focus on granting of study leave to nursing staff and procedures for delegation to attend workshops should be reinforced.

### Nursing education

The issues of unfair labour practices in the healthcare facilities should be included in the nursing curriculum.

### Policymaking

There is need for the Department of Health to consider revising a policy on staff development in order to review the criteria to be followed with regard to delegation of attending supervision and management workshops at the level of PHC.

### Areas for future research

Further studies could focus on the experiences of local health area managers in the PHC facilities with regard to support towards operational managers in the supervision and management of PHC facilities, North West province.

## Data Availability

Data sharing is not applicable to this article as no new data were created or analysed in this study.
